# The Cure of Gall-Stones

**Published:** 1913-05-03

**Authors:** 


### THE CURE OF GALL-STONES.

### An Alternative Diagnostic Method.

It has been known for many years that in a very considerable percentage of those upon whom *post-mortem* examinations are conducted, no matter what the cause of their death, one or more gall-stones will be found present in the gall-bladder. Indeed, for everyone in whom this condition was diagnosed there must have been scores in whom it was never even suspected. To the female sex this statement applied with a good deal more force than to the male; and competent observers have reckoned that 25 per cent. of women, over sixty, have stones in the gall-bladder. Needless to say, in nowhere near such a proportion of elderly women do there exist the classical symptoms of biliary colic, jaundice, and abdominal tumour of a cholecystic type; hence the omission, we cannot call it failure, to discover the condition during life. The natural inferences possible from these given facts were two: one, that the symptoms regarded as typical of gall-stones are really only indicative of an uncommon variety or of complications of simple cholelithiasis; the other, that in most cases gall-stones cause no symptoms at all. With a light heart the latter alternative was adopted; and for several years it was a cardinal article of medical faith that gall-stones may not, and commonly do not, cause any symptoms.

The other alternative, that colic, jaundice, and enlarged gall-bladder are accidents, as it were, in the history of gall-stones, and that the true symptoms which should make the physician suspect this condition are quite other than the three very striking ones just catalogued, has been taken into favour during the last year or two, mainly owing to the teachings and writings of Sir Berkeley Moyniham, of Leeds. According to this view, gall-stones very seldom fail to cause symptoms; the failure to recognise those symptoms is the root of the comparative infrequency with which the disease was formerly diagnosed during life. This conception constitutes a very real advance in diagnosis, and entails a corresponding advance in treatment; for Sir Berkeley is very emphatic that an enormous amount of chronic pain, ill-health, and invalidism can be cut short by removing the stones which he has taught the world how to discover. Following his example, surgeons are operating very freely on the gall-bladder, and in a few years it will be possible to analyse results and to pronounce definitely whether Sir B. Moyniham is, as he seems, right.

Meanwhile, the end-results of gall-stone cases can be studied to a limited degree only; because most of the patients who have been operated on sufficiently long ago to be of value statistically were those who developed some of the old "classical" symptoms, and were therefore in most cases of very old standing. Still, if operation cures these presumably severe cases, so much the more may it be expected to benefit those in whom the mischief is of shorter standing.

A survey of all the gall-stone cases operated on in the Bristol Royal Infirmary during eleven and a half years has been opportunely published by Dr. A. R. Short.* Excluding all cases in which a malignant growth co-existed, eighty-six patients were operated on for gall-stones. Of these, twelve died in the infirmary; this somewhat high proportion is doubtless due to the fact that many of the patients were operated on because of complications. On Moyniham's lines these will become steadily rarer, because the condition will be taken in hand long before the stage at which such complications supervene. It must not be inferred, therefore, that the risk of an operation for gall-stones in ordinary circumstances is anything like so great as this.

This leaves seventy-four survivors, of whom sixty were traced and the other fourteen have been completely lost sight of. Some of the sixty were traced ten years back; others, towards the end of the series, for only fourteen months; none were included under the latter limit. Out of these sixty, thirty are completely and entirely cured without any shadow of doubt or reservation. Considering that many of them are middle-aged or elderly women of the working classes, frequently obese in addition, this is a remarkable result. A further nineteen, though not quite in perfect health, are so greatly improved that they do not need medical treatment and can carry on the ordinary affairs of life. Two have died since the operation of other causes. The remaining nine are not much better off than they were before the operation. The failures are in many cases attributable to the presence of adhesions. When these are present, failures amount to 36 per cent.; when they are not, to only 10 per cent.

It is one of the arguments in favour of early surgical treatment of gall-stones, even if the symptoms caused are not such as to inconvenience the patient very seriously, that the continued presence of stones in the gall-bladder is a strong factor in favouring the development of cancer in that situation. That this is so is unquestionable, and most surgeons are now agreed as to the desirability of treating gall-stones by removal as soon as they have been diagnosed. The same argument as to the ætiology of cancer of the gall-bladder has been also used to support the operation of cholecystectomy (removal of the entire gall-bladder) against the mere removal of the stones. The Bristol cases do not support this view, for among the whole sixty patients there has not been a single case of cancer developing in the gall-bladder. The statistics do certainly give a highly favourable impression of the efficacy of gall-bladder surgery; and since, as has been suggested, even better results may be expected to follow earlier operations, the benefits promised by this particular branch of abdominal surgery are enormous.

* *Bristol Medico-Chirurgical Journal*, April, 1913, p. 34.

